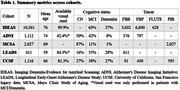# Reproducibility of Centiloid Values in Real‐World Amyloid PET Data: Comparison of the Imaging Dementia‐Evidence for Amyloid Scanning (IDEAS) to Four Large Research Datasets

**DOI:** 10.1002/alz.095827

**Published:** 2025-01-09

**Authors:** Ganna Blazhenets, Ehud Zeltzer, Julien Lagarde, Susan M. Landau, Robert A. Koeppe, Maria C. Carrillo, Bradford C. Dickerson, Liana G. Apostolova, William J. Jagust, Gil D. Rabinovici, Renaud La Joie, Alzheimer’s Disease Neuroimaging Initiative (ADNI)

**Affiliations:** ^1^ Medical Center ‐ University of Freiburg, Faculty of Medicine, University of Freiburg, Freiburg, Baden‐Wuerttemberg Germany; ^2^ University of California, San Francisco, San Francisco, CA USA; ^3^ Department of Neurology, University of California, San Francisco, San Francisco, CA USA; ^4^ University of California, Berkeley, Berkeley, CA USA; ^5^ University of Michigan, Ann Arbor, MI USA; ^6^ Alzheimer's Association, Chicago, IL USA; ^7^ Massachusetts General Hospital and Harvard Medical School, Boston, MA USA; ^8^ Indiana University School of Medicine, Indianapolis, IN USA; ^9^ Lawrence Berkeley National Laboratory, Berkeley, CA USA; ^10^ Weill Institute for Neurosciences, University of California, San Francisco, San Francisco, CA USA; ^11^ Memory and Aging Center, Weill Institute for Neurosciences, University of California, San Francisco, San Francisco, CA USA

## Abstract

**Background:**

The Centiloid framework was developed to harmonize amyloid‐PET quantification across radiotracers and processing pipelines to facilitate data sharing and merging; it is now widely used across research and clinical trials. As we just completed the quantification of 10,361 amyloid‐PET scans from the largest “real‐world” study of amyloid‐PET (IDEAS) and are about to release the data, we aimed to compare the distribution of IDEAS Centiloid values with other available datasets.

**Method:**

In IDEAS, amyloid scans were acquired across 343 facilities and centrally processed at UCSF using a PET‐only pipeline. We also had access to PET data from our own UCSF Alzheimer’s Disease Research Center and the LEADS study. Using the GAAIN platform, we identified two other cohorts with available Centiloids: ADNI and MCSA. For each cohort, we collected Centiloids, demographic, and basic clinical data. Gaussian mixture models (GMM) were fitted to Centiloid values for each cohort, and data‐driven Centiloid cutoffs were calculated as mean + 2SD of the first Gaussian. Finally, we compared Centiloids to PET visual reads (when available) and determined the Centiloid cutoff value maximizing correspondence between visual read and binarized Centiloids based on Cohen’s kappa.

**Result:**

The 5 cohorts were heterogeneous in terms of sample characteristics and radiotracers (Table 1). In all cohorts, a two‐Gaussian model was considered the best fit for the data based on the integrated completed likelihood criteria (Figure 1). The first Gaussian peaks were close to zero, with mild variability across studies (from ‐5 in IDEAS to 10 CL in MSCA). The second peak was more heterogeneous across cohorts (from 67 to 102 CL) with a rightward shift in cohorts enriched with clinically impaired patients. Mean Centiloid values in visually negative and positive scans generally matched well with results derived from GMM (Figure 2). Across all cohorts, GMM‐based Centiloid cutoffs tended to be slightly lower (18‐26) compared to those based on visual inspection (25‐31).

**Conclusion:**

The availability of Centiloids across cohorts enables a direct comparison of amyloid‐PET results in otherwise different studies. Despite some variability across cohorts and analysis methods, Centiloid cutoffs align well with thresholds from the existing literature.